# Electrophysiological readout of ACTH-related neuroendocrine–immunomodulatory treatment response in infantile epileptic spasms syndrome: suppression of scalp ripple propagation networks

**DOI:** 10.3389/fimmu.2026.1923197

**Published:** 2026-08-10

**Authors:** Guoyun Feng, Wentao Dai, Yu Yang, Xianyue Liu, Hua Li, Bingwei Peng, Yuanyuan Che, Xiaonan Li, Jialin Wang, Qianjin Zhou, Jiaqing Yan, Fang Fang, Jian-Min Zhong, Jie Deng, Liemin Zhou, Xiaofeng Yang

**Affiliations:** 1 Guangzhou National Laboratory, Guangzhou, China; 2 Department of Neurology, The Seventh Affiliated Hospital, Sun Yat-sen University, Shenzhen, China; 3 Department of Neurology, The First Affiliated Hospital of Guangzhou Medical University, Guangzhou, China; 4 Department of Neurology, National Center for Children’s Health, Beijing Children’s Hospital, Capital Medical University, Beijing, China; 5 Department of Neurology, Guangzhou Women and Children’s Medical Center, Guangzhou Medical University, Guangzhou, China; 6 Department of Neurology, Jiangxi Provincial Children’s Hospital, Nanchang, China; 7 School of Automation Science and Engineering, South China University of Technology, Guangzhou, China; 8 College of Electrical and Control Engineering, North China University of Technology, Beijing, China

**Keywords:** adrenocorticotropic hormone, developmental and epileptic encephalopathy, electrophysiological response marker, high-frequency oscillations, infantile epileptic spasms syndrome, neuroendocrine–immunomodulatory therapy, ripple propagation network, scalp EEG

## Abstract

**Background:**

Adrenocorticotropic hormone (ACTH) is a first-line neuroendocrine–immunomodulatory therapy for infantile epileptic spasms syndrome (IESS), but noninvasive electrophysiological markers that objectively reflect treatment-related network changes remain limited. We investigated whether scalp putative ripple propagation network metrics provide markers of adrenocorticotropic hormone-related network response and early relapse risk.

**Methods:**

In this retrospective multicenter study, children receiving first-time ACTH therapy were included if paired pre- and post-treatment scalp electroencephalography (EEG) recordings and at least 6 months of follow-up were available. Five-minute artifact-free interictal slow-wave-sleep segments were analyzed. Putative ripple propagation networks were constructed using a time-delay-based method. The primary metrics were post-treatment total network connection strength (total NCS) and its reduction ratio, with ripple count and ripple-count reduction ratio used as rate-based comparators.

**Results:**

Sixty-one children were included; 27 achieved acute spasm freedom, and seven of these relapsed within 6 months. Baseline ripple count and network metrics did not distinguish subsequent responders from nonresponders. After adrenocorticotropic hormone therapy, acute responders showed marked suppression of putative ripple propagation network metrics, whereas nonresponders showed reduction in ripple count without comparable reductions in network metrics. Post-treatment total network connection strength and its reduction ratio differentiated acute responders from nonresponders, with areas under the curve (AUC) of 0.902 and 0.899, respectively. In exploratory analyses among acute responders, greater total network connection strength reduction was associated with sustained 6-month spasm freedom, although relapse-related estimates were limited by the small number of events.

**Conclusion:**

Scalp putative ripple propagation networks provide a noninvasive electrophysiological readout of ACTH-related network modulation in infantile epileptic spasms syndrome. In contrast to ripple count alone, network connection strength captured whether treatment was accompanied by disruption of propagating high-frequency activity. These findings suggest that total NCS and its reduction ratio may serve as candidate markers of adrenocorticotropic hormone-related neuroendocrine–immunomodulatory therapy response, while their relevance to relapse risk requires prospective validation.

## 1 Introduction

Infantile epileptic spasms syndrome (IESS) is a time-critical developmental and epileptic encephalopathy in early life, characterized by epileptic spasms, abnormal developmental trajectories, and abnormal EEG backgrounds, classically including hypsarrhythmia or modified hypsarrhythmia (1, 2). Beyond abnormal cortical excitability alone, accumulating evidence suggests that epileptic encephalopathies may involve complex interactions among developmental neural circuits, neuroendocrine regulation, stress-response pathways, and neuroimmune signaling (2–5). This broader biological framework is particularly relevant to IESS, in which rapid suppression of pathological network activity is essential for seizure control and developmental outcome (6–9). Adrenocorticotropic hormone (ACTH) remains one of the most important first-line therapies for IESS (9, 10). Although traditionally classified as hormonal therapy, ACTH-based treatment can also be viewed as a neuroendocrine–immune intervention, because its proposed mechanisms include hypothalamic–pituitary–adrenal axis regulation, glucocorticoid-responsive signaling, melanocortin receptor pathways, inflammatory modulation, and downstream effects on neuronal excitability (2, 4, 5, 11). However, the mechanisms underlying ACTH responsiveness remain incompletely defined, and clinical outcomes after ACTH therapy are heterogeneous (5, 9, 12). Some children achieve rapid spasm freedom, whereas others fail to respond or relapse after apparent early remission (9, 12, 13). Therefore, objective biomarkers are needed to verify whether ACTH-related neuroendocrine–immune therapy has effectively suppressed the pathological epileptic network.

Current assessment of ACTH response mainly relies on clinical spasm cessation and visual EEG improvement, including resolution of hypsarrhythmia or reduction of epileptiform burden (4, 10, 13, 14). These measures are clinically essential but may not fully capture whether pathological high-frequency network activity has been disrupted. Several quantitative EEG biomarkers have been investigated in IESS, including the Burden of Amplitudes and Epileptiform Discharges score, spectral power, entropy, long-range temporal correlations, functional connectivity, phase–amplitude coupling, and high-frequency oscillations (6, 7, 12, 13, 15–20). However, most of these approaches primarily quantify abnormal activity burden or synchrony, rather than directly measuring whether high-frequency epileptic activity remains organized as a propagating network after treatment.

High-frequency oscillations, including ripples and fast ripples, are increasingly recognized as electrophysiological markers of epileptogenic tissue and pathological network excitability (21–24). Scalp-recorded HFOs have been reported in children with epileptic encephalopathies and have been associated with disease activity, treatment response, prognosis, and EEG severity in IESS (19, 25–29). Nevertheless, most prior scalp HFO studies have focused on event rate, amplitude, power, or spatial distribution. These rate-based measures address how many HFO events occur, but they do not determine whether residual HFOs continue to participate in a temporally organized propagation network. This distinction is important because ACTH-related clinical improvement may require not only reduction of local high-frequency events but also disruption of the propagation architecture that supports pathological epileptic network activity.

We previously developed a time-delay-based approach for constructing directed HFO propagation networks from electrophysiological recordings (30). In the present multicenter retrospective study, we adapted this framework to routine scalp EEG in children with IESS receiving first-time ACTH therapy. We tested whether suppression of scalp ripple propagation network strength could serve as a noninvasive neuroelectrophysiological biomarker for verifying response to ACTH-related neuroendocrine–immune therapy, and whether residual ripple propagation after apparent acute response was associated with early relapse. We hypothesized that effective ACTH therapy would be accompanied by marked suppression of pathological ripple propagation networks beyond simple ripple-count reduction. This study was not designed to prove a direct immunological mechanism, but to evaluate scalp HFO network suppression as a downstream electrophysiological readout of ACTH-related treatment response.

## 2 Materials and methods

### 2.1 Study design and participating centers

This retrospective multicenter cohort study was conducted at three pediatric epilepsy centers in China: Beijing Children’s Hospital, Capital Medical University; Guangzhou Women and Children’s Medical Center, Guangzhou Medical University; and Jiangxi Provincial Children’s Hospital. Eligible cases were identified from institutional epilepsy databases and medical records between January 2017 and July 2024, with center-specific start dates reflecting database availability.

The study was approved by the institutional review board or ethics committee at each participating center. Given the retrospective design and use of deidentified clinical and EEG data, the requirement for written informed consent was waived. Clinical data extraction, EEG preprocessing, ripple detection, network construction, and statistical analysis were harmonized across centers. Investigators performing computational EEG analysis were blinded to clinical outcomes.

### 2.2 Patient selection

Inclusion criteria were (1) diagnosis of IESS according to contemporary ILAE syndrome classification; (2) first-time ACTH therapy administered intravenously at 1–4 IU/kg/day for 14 consecutive days; (3) available pre-treatment scalp EEG recorded within 15 days before ACTH initiation and post-treatment scalp EEG recorded within 7 days after completion of ACTH; (4) EEG recordings containing artifact-free interictal slow-wave sleep segments suitable for ripple and network analysis; and (5) clinical follow-up for at least 6 months after ACTH therapy.

Patients were excluded if they had: (1) adjustment of anti-seizure medications during the ACTH treatment window; (2) absence of daily epileptic spasms before treatment; (3) missing or poor-quality EEG data precluding ripple or network analysis; (4) prior or concurrent hormonal therapy during the study window; or (5) incomplete clinical or follow-up data.

### 2.3 EEG acquisition and preprocessing

Scalp EEG recordings were acquired before and after ACTH treatment. Each recording lasted at least 2 hours to ensure adequate sampling of interictal slow-wave sleep. EEGs were recorded using Nicolet EEG systems (Natus Medical Incorporated, Middleton, WI, USA) or Nihon Kohden EEG systems (Nihon Kohden Corporation, Tokyo, Japan). Electrodes were placed according to the International 10–20 system. Original sampling rates ranged from 1000 to 2000 Hz. To standardize data across centers and recording platforms, all EEG signals were downsampled to 1000 Hz using a polyphase anti-aliasing filter in MATLAB R2022b (MathWorks, Natick, MA, USA).

For each recording, a continuous 5-minute interictal segment from the first slow-wave sleep cycle was selected. Slow-wave sleep was defined as a 30-second epoch containing more than 25% delta activity, consistent with prior scalp HFO studies (26, 27). Segments containing visually evident noncerebral contamination, including movement-related transients, myogenic high-frequency activity, electrode-related disturbances, or filter ringing associated with sharp transients, were excluded by a board-certified pediatric electroencephalographer according to standard clinical EEG and HFO evaluation recommendations (31, 32).

EEG preprocessing was performed using EEGLAB and custom MATLAB scripts. Electromyography and electrocardiography channels were removed. Nineteen standard scalp EEG channels were retained and re-referenced to a longitudinal bipolar montage, yielding 18 fixed bipolar channels for network analysis. The bipolar derivations were Fp1–F7, F7–T3, T3–T5, T5–O1, Fp2–F8, F8–T4, T4–T6, T6–O2, Fp1–F3, F3–C3, C3–P3, P3–O1, Fp2–F4, F4–C4, C4–P4, P4–O2, Fz–Cz, and Cz–Pz. Power-line interference and its harmonics were removed using Hamming-windowed linear-phase FIR notch filters centered at 50, 100, 150, and 200 Hz, with stopbands of 47–53 Hz, 97–103 Hz, 148–152 Hz, and 197–203 Hz, respectively. Signals were subsequently filtered between 80 and 200 Hz to isolate ripple-band activity. The same preprocessing pipeline was applied to all recordings.

### 2.4 Automatic scalp ripple detection

Scalp ripples were detected using a previously validated automatic HFO algorithm based on the maximum-distributed-peak-point method (33). The algorithm used overlapping 30-s windows with a 1-s step and four amplitude thresholds to identify ripple events. Events were marked as ripples if they contained at least 8 consecutive peak points above the low threshold and at least 6 peak points above the high threshold (26, 27). Thresholds were defined relative to baseline amplitude within each moving window. The same detection thresholds and analysis parameters were applied across all centers.

Ripple count was defined as the total number of automatically detected ripple events within the 5-minute slow-wave sleep segment. Continuous 5-minute slow-wave sleep epochs have been used in prior HFO studies and were selected here to balance biological sampling, clinical feasibility, and artifact control (34). The reduction ratio (RR) for each metric was calculated as:


$$RR=\frac{N_{pre}-N_{post}}{N_{pre}}$$

where *N_pre_* and *N_post_* represent pre-treatment and post-treatment values, respectively. A positive reduction ratio indicates post-treatment reduction of the corresponding metric, whereas a negative value indicates an increase relative to baseline. No zero pre-treatment values were observed for ripple count, network node number, total NCS or mean NCS; therefore, no additional handling for zero denominators was required.

Ripple count and ripple-count reduction ratio served as comparator metrics for network-based analyses.

### 2.5 Ripple propagation network construction

**Figure 1:**
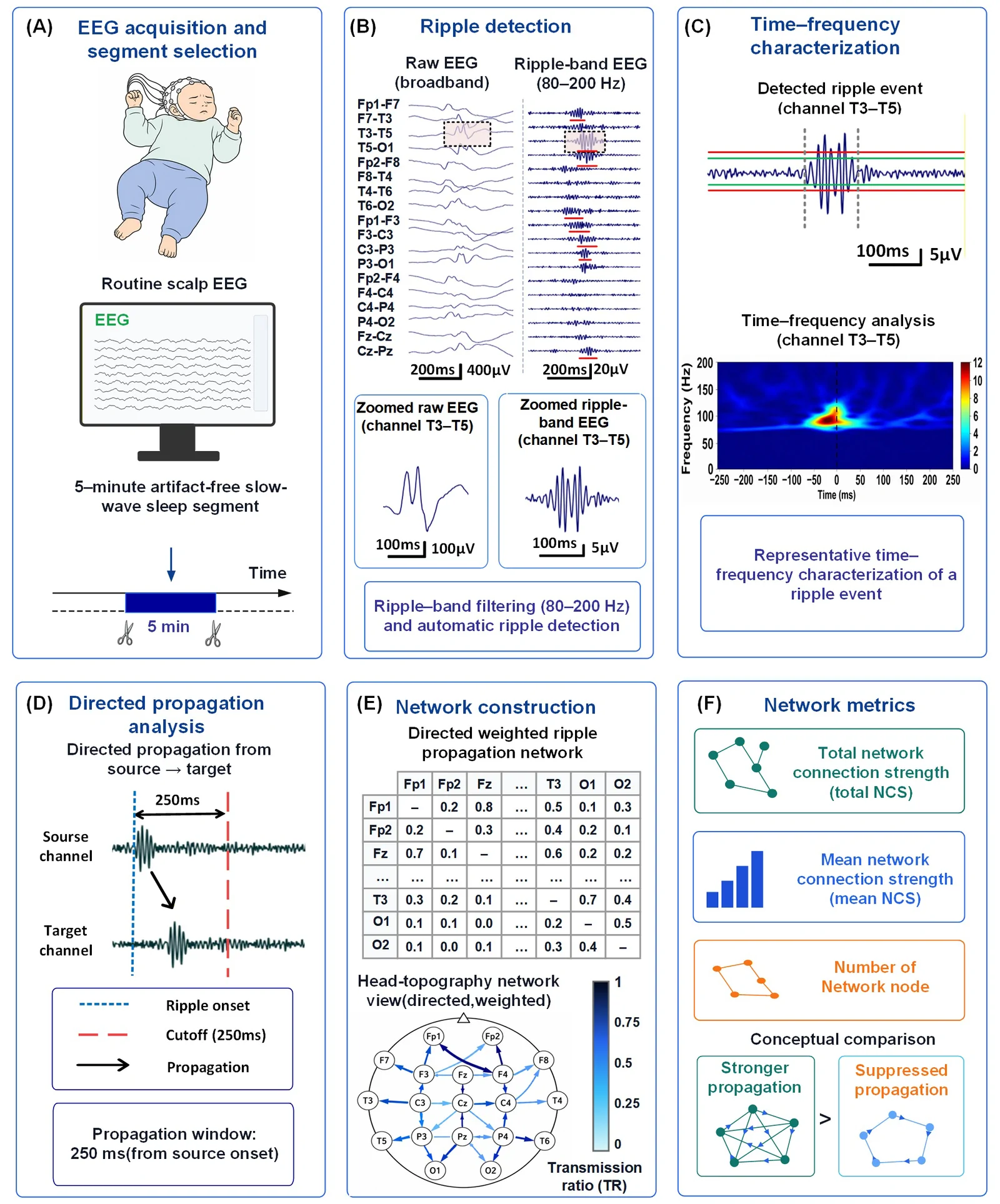
Workflow for constructing scalp ripple propagation networks from routine electroencephalography. **(A)** A 5-minute artifact-free interictal slow-wave-sleep segment was selected from routine scalp electroencephalography (EEG). **(B)** Broadband EEG was filtered into the 80–200 Hz ripple band, and ripple events were automatically detected across bipolar channels. Zoomed broadband and ripple-band traces illustrate the morphology of a representative detected event. **(C)** Representative time–frequency characterization of a detected ripple event. The Morlet wavelet time–frequency map demonstrates a localized narrow-band power increase within the ripple frequency range (80–200 Hz), corresponding to the oscillatory activity observed in the ripple-band filtered signal. **(D)** Directed propagation was defined as a time-lagged ripple pair in which a ripple first occurred in the source channel and was followed by another ripple in the target channel within a 250-ms window. The blue dashed line marks the source-channel ripple onset, the red long-dashed line marks the 250-ms cutoff, and the black arrow indicates putative propagation from source to target. **(E)** Pairwise TR values were calculated to generate a directed weighted adjacency matrix and a scalp topographic ripple propagation network. The color bar indicates TR values from 0 to 1, with darker edges representing stronger propagation. **(F)** Three network metrics were derived: total NCS, mean NCS (node-normalized total NCS, calculated as total NCS divided by the number of participating network nodes), and network node number. These metrics were used to characterize treatment-related changes in high-frequency propagation networks after adrenocorticotropic hormone therapy. EEG, electroencephalography; TR, transmission ratio; NCS, network connection strength.

Directed ripple propagation networks were constructed using a time-delay-based approach adapted from prior HFO network work (30), as summarized in **Figure 1**. For each patient and each EEG session, all 18 × 17 ordered bipolar channel pairs were evaluated. A directed channel pair from channel A to channel B represented potential propagation from source channel A to target channel B.

For each source-channel ripple, the propagation window was defined as the 250-ms interval beginning at the source ripple onset. This delay was prespecified before analysis of the clinical outcomes and was retained from our previously published time-delay-based HFO network framework (30), consistent with the temporal-association window used in prior HFO network research (35). It was used as an operational interval for identifying candidate time-lagged ripple associations and should not be interpreted as a direct estimate of physiological propagation delay. If the onset of a target-channel ripple occurred within this 250-ms window, the event was counted as a candidate propagation event from the source to the target channel.

The directed transmission ratio from source channel s to target channel t was calculated as:


$$TR_{s\to t}=\frac{n_{s\to t}}{N_{t}}$$

where $n_{s\to t}\;$is the number of target-channel ripple events preceded by a source-channel ripple within the predefined propagation window, and $N_{t\;}$is the total number of ripples detected in the target channel. However, a single target-channel ripple could be associated with multiple source channels when ripples were detected in more than one source channel within the corresponding window, with each source – target pair evaluated independently.

This target-normalized ratio estimates the proportion of target-channel ripple events that can be temporally attributed to a preceding ripple in the source channel. It should therefore be interpreted as an incoming explanatory fraction of the target channel rather than as the outgoing propagation efficiency of the source channel.

To reduce unstable or spurious connections, two quality-control criteria were applied. First, if either channel contained fewer than two ripples, the TR for that ordered pair was set to zero. Second, the ripple-count ratio for the directed connection s→t was calculated as $N_{s}/N_{t}$, where $N_{s}$ and $N_{t}$ are the total numbers of ripples detected in the source and target channels, respectively. If $N_{s}/N_{t}<0.5$, the corresponding $TR_{s\to t}$ was set to zero to avoid connections driven by highly imbalanced ripple distributions. Because this criterion was directional, the reverse connection t→s was evaluated independently using $N_{t}/N_{s}$. For each EEG segment, an 18 × 18 directed TR matrix was generated.

### 2.6 Network metrics

Channels without any sent or received propagation were excluded from the final network. Three network-level metrics were extracted:

1. Number of network nodes: the number of bipolar channels participating in at least one directed propagation.
2. Total network connection strength (total NCS): the sum of all non-zero directed TR values within the network.
3. Node-normalized total NCS (referred to as mean NCS hereafter): calculated as the total NCS divided by the number of participating network nodes. This metric represents the average network connectivity contribution per participating node and differs from conventional mean edge weight, which is calculated by averaging the weights of all non-zero connections.

The primary network metrics were total NCS and total NCS reduction ratio. Mean NCS, node number, and their reduction ratios were considered secondary exploratory network metrics.

### 2.7 Clinical variables and outcome definitions

Clinical variables included sex, age at spasm onset, etiology, disease duration before ACTH therapy, number of anti-seizure medications at ACTH initiation, and daily spasm frequency before ACTH treatment. Etiology was categorized as genetic, metabolic, structural, or unknown according to clinical evaluation. Data were extracted from electronic medical records by investigators blinded to EEG-derived metrics.

The primary clinical outcome was acute spasm freedom after ACTH therapy. All participants underwent post-treatment video-EEG examination within 7 days after completion of ACTH. Acute spasm freedom was defined as complete cessation of clinically observed epileptic spasms during this post-treatment period, corroborated by the absence of electroclinical spasms on video-EEG. Patients with persistent clinical or electroclinical spasms were classified as nonresponders.

The secondary clinical outcome was 6-month sustained spasm freedom among acute responders. Relapse was defined as recurrence of epileptic spasms during the 6-month follow-up period. Because relapse analysis was restricted to acute responders and included only seven relapse events, this analysis was considered exploratory.

### 2.8 Statistical analysis

Continuous variables were assessed for normality using the Shapiro–Wilk test. Normally distributed variables were presented as mean ± standard deviation and compared using Student’s t test, whereas non-normally distributed variables were presented as median and interquartile range and compared using the Mann–Whitney U test. Categorical variables were summarized as counts and percentages and compared using chi-square tests or Fisher’s exact tests, with Yates’ correction applied when appropriate.

For paired comparisons, normality was assessed based on the within-subject differences (post-treatment minus pretreatment) using the Shapiro–Wilk test. Paired-samples t tests were performed when the paired differences were normally distributed; otherwise, Wilcoxon signed-rank tests were applied.

Receiver operating characteristic (ROC) curve analyses were performed to evaluate the discriminative performance of ripple-count and network-based metrics for acute ACTH response and 6-month outcomes. The area under the ROC curve (AUC) and corresponding 95% confidence intervals were calculated. Optimal cut-off values were determined using the Youden index. Sensitivity, specificity, accuracy, positive predictive value, negative predictive value, positive likelihood ratio, and negative likelihood ratio were calculated for selected thresholds.

To evaluate the robustness of discrimination performance, repeated 10 × 5-fold cross-validation was performed using the caret package in R under fixed random seed conditions. Folds were stratified according to outcome whenever possible. AUC values obtained from each cross-validation repetition were retained for statistical comparison. Paired comparisons of cross-validation-derived AUC values were performed using paired-samples t tests or Wilcoxon signed-rank tests according to the distribution of paired AUC differences.

Multiple comparisons were corrected according to prespecified comparison families. The Holm procedure was used for primary confirmatory comparisons to control the family-wise error rate, whereas the Benjamini–Hochberg procedure was applied to secondary exploratory comparisons to control the false discovery rate. All statistical tests were two-tailed, and adjusted P values <0.05 were considered statistically significant.

## 3 Results

### 3.1 Patient cohort

A total of 101 children met the initial screening criteria. Forty were excluded because of poor-quality or missing EEG data (n = 14), anti-seizure medication changes during the ACTH treatment window (n = 19), absence of daily spasms before treatment (n = 3), previous or concurrent hormonal therapy (n = 1), or incomplete clinical data (n = 3). Sixty-one children were included in the final analysis.

After ACTH therapy, 27 children achieved acute spasm freedom and 34 did not. Among the 27 acute responders, 20 remained spasm-free and 7 relapsed during the 6-month follow-up period. Demographic and clinical variables did not differ significantly between acute responders and nonresponders, nor between non-relapse and relapse subgroups among acute responders (all raw P > 0.05; **Table 1**). Patient selection is summarized in **Supplementary Figure 1**.

**Table 1:** Demographic and clinical characteristics.

Characteristic	Acute responders (N = 27)		Nonresponders (N = 34)
	Non-relapse (N = 20)	Relapse (N = 7)	
Sex			
Male	12 (60)	2 (28.6)	15 (44.1)
Female	8 (40)	5 (71.4)	19 (55.9)
Number of ASMs			
1	11 (55)	5 (71.4)	13 (38.2)
≥2	9 (45)	2 (28.6)	21 (61.8)
Etiology			
Genetic	1 (5)	0 (0)	6 (17.6)
Metabolic	1 (5)	0 (0)	2 (5.9)
Structural	5 (25)	2 (28.6)	10 (29.4)
Unknown	13 (65)	5 (71.4)	16 (47.1)
Age at onset (m)	6.5 (3.0, 8.3)	4.0 (3.0, 6.0)	5.0 (3.0, 8.0)
Course of the disease (m)	1.0 (1.0, 2.0)	3.0 (2.0, 7.0)	3.5 (1.0, 7.3)
Daily seizure frequency pre-ACTH	4.0 (2.6, 5.8)	2.3 (2.0, 6.0)	5.1 (3.0, 8.5)

Categorical variables are presented as n (%), and continuous variables as median (IQR). ASMs, anti-seizure medications; m, months; ACTH, adrenocorticotropic hormone.

### 3.2 Acute ACTH response

#### 3.2.1 Baseline ripple and network metrics before ACTH therapy

**Figure 2:**
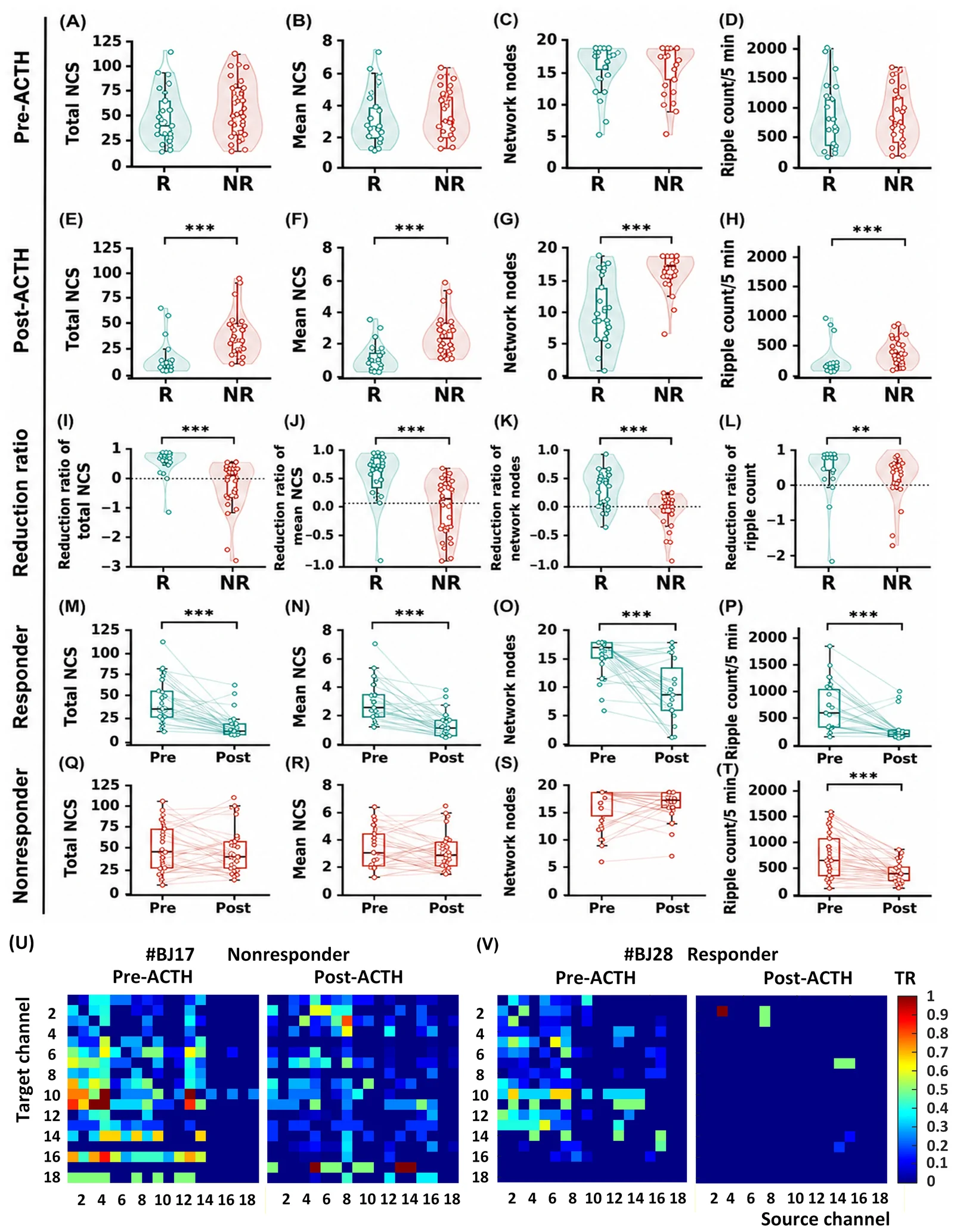
ACTH-related changes in scalp ripple propagation network metrics between acute responders and nonresponders. **(A–D)** Pre-treatment comparisons of total NCS, mean NCS, network node number, and ripple count/5 min between acute responders and nonresponders. For simplicity, acute responders and nonresponders are denoted as R and NR, respectively, in the figures. **(E–H)** Post-treatment comparisons of the same metrics between acute responders and nonresponders. **(I–L)** Between-group comparisons of reduction ratios for total NCS, mean NCS, network node number, and ripple count. **(M–P)** Within-group pre- and post-treatment comparisons in acute responders. **(Q–T)** Within-group pre- and post-treatment comparisons in nonresponders. Violin plots show data distributions, boxplots indicate medians and interquartile ranges, and individual points represent individual patients. Lines in paired plots connect pre- and post-treatment values from the same patient. **(U, V)** Representative directed, weighted ripple propagation network matrices before and after adrenocorticotropic hormone therapy for a nonresponder and a responder, respectively. Rows and columns indicate target and source bipolar channels, respectively. Each matrix element represents the directed propagation strength (TR value) from the source channel (column) to the target channel (row). Channel numbers correspond to the predefined bipolar channel order: Fp1–F7, F7–T3, T3–T5, T5–O1, Fp2–F8, F8–T4, T4–T6, T6–O2, Fp1–F3, F3–C3, C3–P3, P3–O1, Fp2–F4, F4–C4, C4–P4, P4–O2, Fz–Cz, and Cz–Pz. Color intensity indicates the magnitude of TR values, with higher values representing stronger directed transmission relationships. *Adjusted P < 0.05, **adjusted P < 0.01, ***adjusted P < 0.001. ACTH, adrenocorticotropic hormone; NCS, network connection strength; TR, transmission ratio.

Before ACTH therapy, no significant differences were observed between acute responders and nonresponders in total NCS (median 32.87, IQR 22.19–57.61 vs. 42.63, IQR 23.07–71.24; adjusted P = 0.523; **Figure 2A**), mean NCS, network node number, and ripple count (all adjusted P = 0.523; **Figures 2B–D**), indicating that baseline scalp ripple burden and network connectivity did not distinguish subsequent treatment response.

#### 3.2.2 Post-ACTH network suppression in acute responders

After ACTH therapy, acute responders showed substantially lower network metrics than nonresponders. Post-treatment total NCS was lower in responders than in nonresponders (median 4.02, IQR 0.75–12.51 vs. 36.21, IQR 21.38–54.57; adjusted P < 0.001; **Figure 2E**). Mean NCS, network node number, and ripple count were also significantly lower in responders than in nonresponders after therapy (all adjusted P < 0.001; **Figures 2F–H**).

Reduction ratios demonstrated a similar pattern. Total NCS reduction ratio was higher in responders than in nonresponders (median 0.88, IQR 0.67–0.97 vs. 0.18, IQR −0.59–0.43; adjusted P < 0.001; **Figure 2I**). Reduction ratios for mean NCS, network node number, and ripple count were also higher in responders (all adjusted P < 0.001; **Figures 2J–L**).

Within-group comparisons showed that acute responders had significant post-treatment reductions in total NCS, mean NCS, network node number, and ripple count (all adjusted P < 0.001; **Figure 2M–P**). In contrast, nonresponders showed a significant reduction in ripple count (adjusted P < 0.001; **Figure 2T**) but no significant reductions in total NCS (adjusted P = 0./348; **Figure 2Q**), mean NCS (adjusted P = 0.348; **Figure 2R**), or network node number (adjusted P = 0.874; **Figure 2S**). Representative heatmaps illustrated marked reductions in putative ripple propagation patterns in an acute responder and persistent network connectivity in a nonresponder (**Figures 2U, V**).

#### 3.2.3 Differentiation of acute ACTH response

ROC analysis showed that post-treatment total NCS had the highest AUC for discriminating acute responders from nonresponders (AUC 0.902, 95% CI 0.799–0.963). Total NCS reduction ratio showed similar differentiation (AUC 0.899, 95% CI 0.794–0.961). Ripple count and ripple-count reduction ratio had lower AUCs of 0.821 (95% CI 0.702–0.907) and 0.769 (95% CI 0.643–0.867), respectively (**Supplementary Table 1**).

At the optimal threshold, post-treatment total NCS showed a sensitivity of 94.1%, specificity of 85.2%, and accuracy of 90.2%. The total NCS reduction ratio showed a sensitivity of 94.1%, specificity of 81.5%, and accuracy of 88.5%. Detailed diagnostic performance metrics are provided in **Supplementary Table 1**.

**Figure 3:**
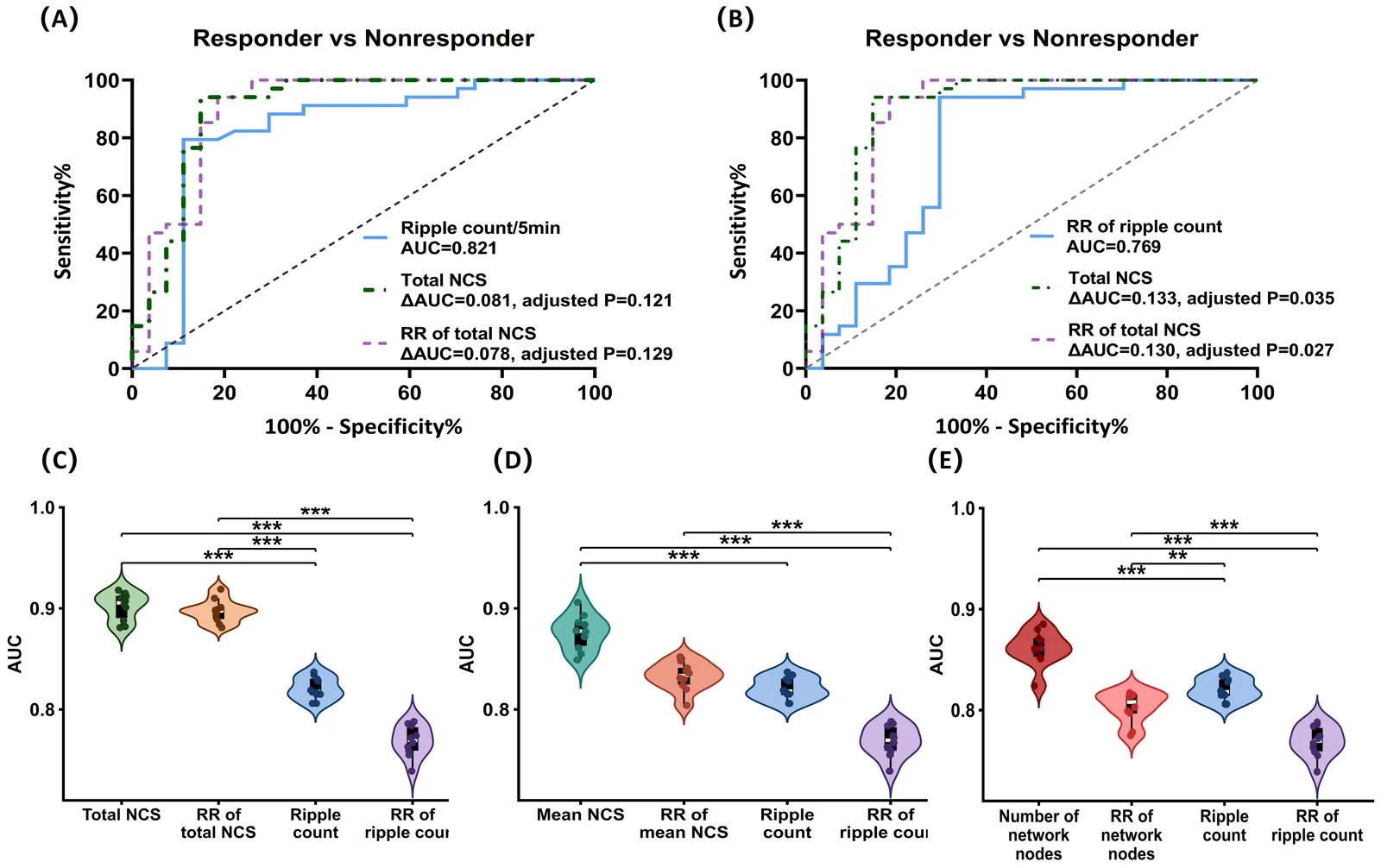
Differentiation and cross-validated stability of ripple-derived metrics for acute ACTH response. **(A, B)** ROC curves comparing ripple count, ripple-count reduction ratio, total NCS, and total NCS reduction ratio for discriminating acute responders from nonresponders. ΔAUC and adjusted P values indicate differences in AUC compared with ripple count metrics. The blue solid line denotes ripple count/5 min in **(A)** and ripple-count reduction ratio in **(B)**; the green dash-dotted line denotes total NCS; and the purple dashed line denotes RR of total NCS. **(C)** Repeated 10 × 5-fold cross-validated AUC distributions for primary network metrics compared with ripple-count metrics. **(D, E)** Repeated 10 × 5-fold cross-validated AUC distributions for secondary network metrics compared with ripple-count metrics. AUC, area under the curve; RR, reduction ratio; ROC, receiver operating characteristic curve; NCS, network connection strength. **adjusted P < 0.01, ***adjusted P < 0.001.

Pairwise DeLong testing showed that total NCS and total NCS reduction ratio were numerically higher than ripple count, but these differences did not reach statistical significance (ΔAUC = 0.081, adjusted P = 0.121 P; ΔAUC = 0.078, adjusted P = 0.129, **Figure 3A**). By contrast, total NCS and total NCS reduction ratio both showed significantly greater differentiation than ripple-count reduction ratio (ΔAUC = 0.133, adjusted P = 0.035; ΔAUC = 0.130, adjusted P = 0.027, respectively; **Figure 3B**). Secondary network metrics showed intermediate AUC values and are reported in **Supplementary Tables 2** and **3**.

#### 3.2.4 Internal resampling stability for acute-response differentiation

Repeated 10 × 5-fold internal resampling suggested that the differentiation estimates for total NCS and total NCS reduction ratio were relatively stable within this cohort. Median cross-validated AUCs were 0.906 (IQR 0.887–0.913) for total NCS and 0.898 (IQR 0.888–0.903) for total NCS reduction ratio. By comparison, median cross-validated AUCs were 0.818 (IQR 0.813–0.831) for ripple count and 0.770 (IQR 0.758–0.785) for ripple-count reduction ratio.

Both primary network metrics showed higher cross-validated AUCs than ripple count and ripple-count reduction ratio (all adjusted P < 0.001) (**Figure 3C**). Among secondary network metrics, mean NCS and network node number also showed higher cross-validated performance than ripple-count reduction ratio, although these analyses were considered exploratory (all adjusted P < 0.01, **Figures 3D, E**).

### 3.3 Six-month outcome

#### 3.3.1 Ripple and network metrics in 6-month non-relapse versus relapse subgroups

**Figure 4:**
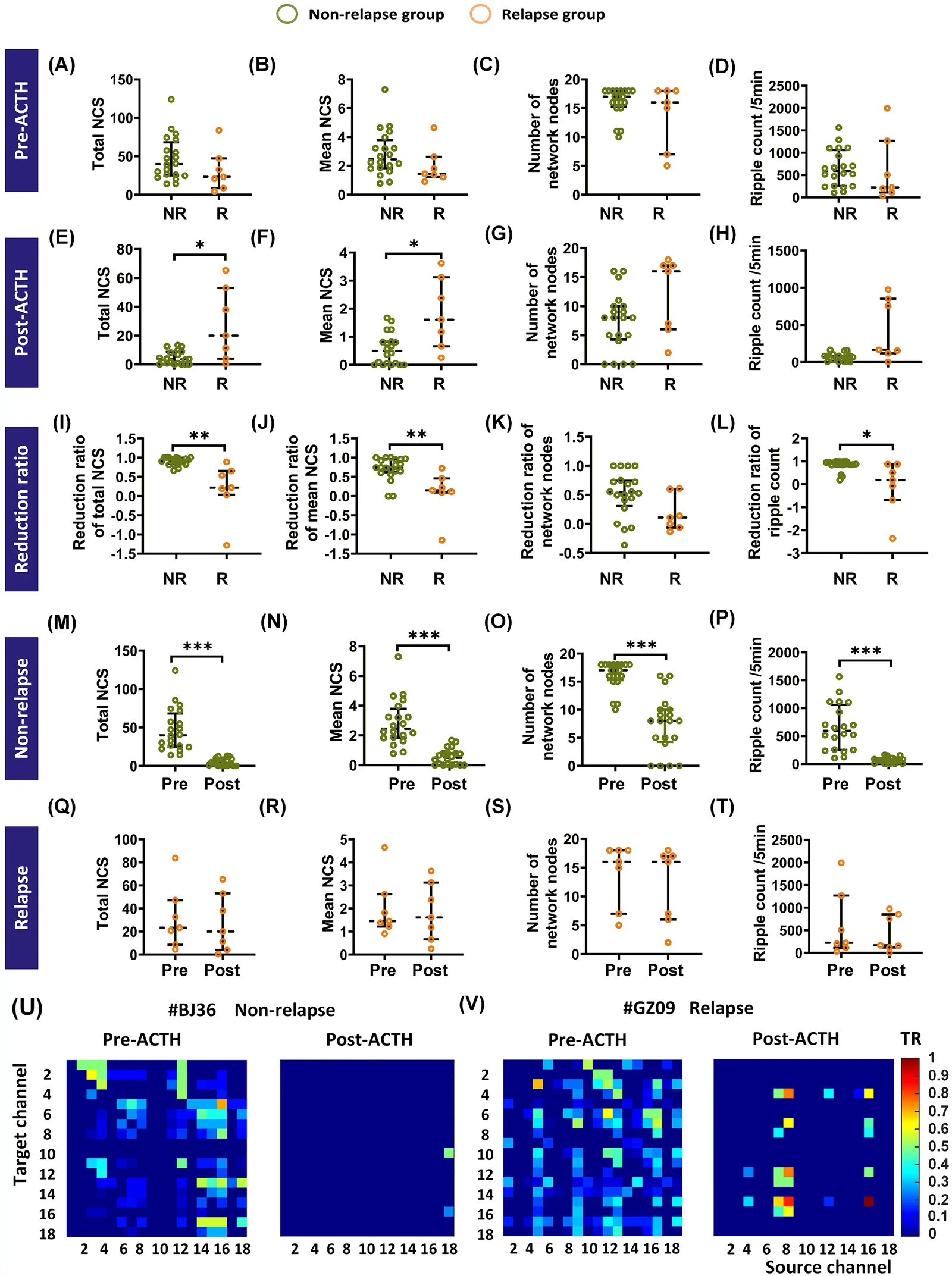
Ripple count and propagation network metrics in sustained spasm freedom and relapse subgroups. **(A–H)** Baseline and post-treatment total NCS, mean NCS, network node number, and ripple count in acute responders who remained spasm-free or relapsed during 6-month follow-up. (For simplicity, relapse and non-relapse are denoted as R and NR, respectively, in the figures.) **(I–L)** Reduction ratios. **(M–T)** Within-group pre/post comparisons in non-relapse **(M–P)** and relapse **(Q-T)** groups. **(U, V)** Representative directed ripple propagation heatmaps of TR matrix from sustained-response and relapse cases. Relapse-related analyses are exploratory because only seven relapse events were observed. Rows and columns indicate target and source bipolar channels, respectively. Each matrix element represents the directed propagation strength (TR value) from the source channel (column) to the target channel (row). Channel numbers correspond to the predefined bipolar channel order: Fp1–F7, F7–T3, T3–T5, T5–O1, Fp2–F8, F8–T4, T4–T6, T6–O2, Fp1–F3, F3–C3, C3–P3, P3–O1, Fp2–F4, F4–C4, C4–P4, P4–O2, Fz–Cz, and Cz–Pz. Color intensity indicates the magnitude of TR values, with higher values representing stronger directed propagation. ACTH, adrenocorticotropic hormone; NCS, network connection strength; TR, transmission ratio. *Adjusted P < 0.05, **adjusted P < 0.01, ***adjusted P < 0.001.

Among the 27 acute responders, 20 maintained spasm freedom and 7 relapsed during 6-month follow-up. Before ACTH therapy, relapse and non-relapse subgroups did not differ significantly in total NCS, mean NCS, network node number, or ripple count (adjusted P = 0.301, 0.301, 0.585, 0.391, respectively; **Figures 4A–D**).

After ACTH therapy, children who remained spasm-free had lower total NCS than those who relapsed (adjusted P = 0.034; **Figure 4E**). Mean NCS also differed between groups (adjusted P = 0.034; **Figure 4F**). Post-treatment network node number and ripple count showed trends but did not reach statistical significance (adjusted P = 0.073 and adjusted P = 0.073, respectively; **Figures 4G, H**).

Reduction ratios were higher in the non-relapse group for total NCS (median 0.90, IQR 0.84–0.99 vs. 0.22, IQR 0.03–0.65; adjusted P = 0.003; **Figure 4I**), mean NCS (adjusted P = 0.009; **Figure 4J**), and ripple count (adjusted P = 0.015; **Figure 4L**). Network node reduction ratio did not differ significantly between groups (adjusted P = 0.060; **Figure 4K**). Within-group analyses showed significant post-treatment reductions in all four metrics among children with sustained spasm freedom (all adjusted P < 0.001, **Figures 4M–P**), whereas no significant post-treatment reductions were observed in the relapse subgroup (adjusted P = 0.545, 0.533, 0.533, 0.545, respectively; **Figures 4Q–T**). Representative heatmaps illustrated corresponding differences in putative ripple propagation patterns (**Figures 4U, V**).

#### 3.3.2 Exploratory association of 6-month outcome

In the exploratory relapse analysis, total NCS reduction ratio showed the highest differentiation for relapse versus sustained spasm freedom (AUC 0.943, 95% CI 0.781–0.996). Reduction ratio of mean NCS and ripple-count reduction ratio showed AUCs of 0.864 (95% CI 0.678–0.965) and 0.829 (95% CI 0.635–0.945), respectively. Post-treatment mean NCS and total NCS showed lower AUCs of 0.821 and 0.807, respectively (**Supplementary Table 4**)

Using the Youden index, total NCS reduction ratio yielded a Youden-derived exploratory threshold of 0.661. (detailed metrics are provided in **Supplementary Table 4**).

**Figure 5:**
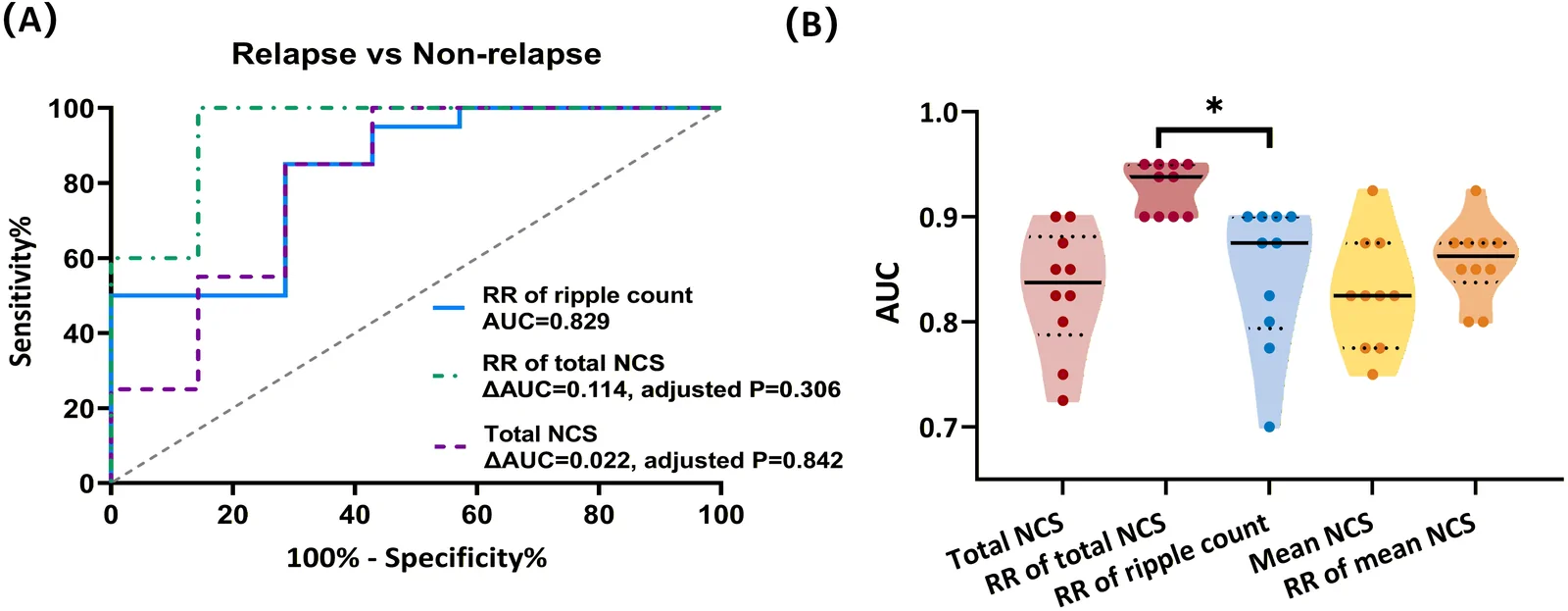
Exploratory discrimination of 6-month relapse status among acute ACTH responders. **(A)** ROC curves for ripple-count reduction ratio (blue solid line), total NCS (purple dashed line), and total NCS reduction ratio (green dash-dotted line) in discriminating sustained spasm freedom from relapse among acute responders. ΔAUC and adjusted P values indicate differences in AUC compared with RR of ripple count. **(B)** Cross-validated AUC distributions for ripple-count and network metrics. Because this analysis included only seven relapse events and was performed within the same retrospective cohort, these ROC and cross-validation results should be interpreted as exploratory and should not be used to define clinical decision thresholds. AUC, area under the curve; RR, reduction ratio; ROC, receiver operating characteristic curve; NCS, network connection strength. *Adjusted P < 0.05.

DeLong testing did not show statistically significant differences between total NCS reduction ratio and ripple-count reduction ratio for 6-month outcome (ΔAUC = 0.114, adjusted P = 0.306; **Figure 5A**, **Supplementary Table 5**). However, repeated cross-validation showed that total NCS reduction ratio had more stable performance than ripple-count reduction ratio (median AUC 0.938 vs. 0.875; adjusted P = 0.019; **Figure 5B**).

## 4 Discussion

The major contribution of this multicenter retrospective study is the identification of scalp ripple propagation network suppression as a noninvasive neuroelectrophysiological readout of ACTH-related neuroendocrine–immunomodulatory therapy response in infantile epileptic spasms syndrome. Effective ACTH therapy was accompanied by marked suppression of putative ripple propagation network metrics, whereas nonresponders showed reduced ripple counts without comparable disruption of network connection strength. This dissociation suggests that reduction in HFO burden and suppression of propagating high-frequency network architecture represent related but non-identical electrophysiological consequences of therapy. Among acute responders, residual ripple propagation showed an exploratory association with early relapse, but this finding should be interpreted as hypothesis-generating because of the small number of relapse events.

### 4.1 From HFO burden reduction to verification of pathological network suppression

Prior scalp HFO studies in IESS have mainly quantified event rate, amplitude, power, spatial distribution, or coupling with slower oscillations (19, 27, 29, 36). These measures are valuable for estimating abnormal high-frequency activity burden, but they do not determine whether residual HFOs remain organized as a directed propagation network. Recent work has increasingly conceptualized HFOs not only as local events but also as markers of epileptogenic network organization (7, 28, 35, 37–39). In the present study, nonresponders showed a significant reduction in ripple count but no significant reduction in total NCS, mean NCS, or network node number. This finding suggests that detectable HFO burden may decrease even when abnormalities in the organization of putative ripple propagation networks remain detectable. Therefore, scalp ripple propagation network analysis may provide information beyond ripple counting by verifying whether treatment has disrupted the high-frequency network organization that supports epileptic spasms.

### 4.2 Ripple propagation suppression as a downstream readout of ACTH-related neuroendocrine–immunomodulatory therapy

ACTH-based treatment is traditionally classified as hormonal therapy for IESS, but its proposed mechanisms involve interacting neuroendocrine, glucocorticoid-responsive, melanocortin, anti-inflammatory, and excitability-modulating pathways (2, 4, 40, 41). Within this framework, suppression of scalp ripple propagation networks may represent a downstream neuroelectrophysiological correlate of effective ACTH-related therapy. The present findings do not prove that ACTH response was mediated by a specific immune pathway. Inflammatory cytokines, peripheral immune-cell profiles, blood–brain barrier or neurovascular-unit markers, and related molecular readouts were not measured. Thus, scalp ripple propagation suppression should be interpreted as a treatment-response readout rather than mechanistic proof of neuroimmune modulation. Future studies combining HFO network metrics with inflammatory, endocrine, imaging, and developmental biomarkers may clarify how ACTH-related biological effects translate into network-level seizure control (40–42).

### 4.3 Clinical relevance: objective verification of ACTH response

Clinical response to ACTH is usually assessed by cessation of spasms and improvement of visual EEG abnormalities, including resolution or improvement of hypsarrhythmia and epileptiform burden (9, 13, 15, 43). However, early post-treatment EEG interpretation can remain challenging, particularly when epileptiform discharges or incomplete background improvement persist. A quantitative HFO propagation metric may add a complementary dimension by measuring whether pathological high-frequency activity has become less connected and less capable of spreading across scalp-recorded networks. In this study, post-treatment total NCS and its reduction ratio showed strong discrimination of acute spasm freedom and outperformed ripple-count reduction ratio. These results support the concept that post-treatment network suppression, rather than baseline network severity alone, may serve as an objective marker for verifying ACTH-related therapeutic effect.

### 4.4 Residual ripple propagation after apparent response: exploratory signal of incomplete network control

Relapse after initial ACTH response remains a major clinical challenge in IESS (13, 15). Previous studies have suggested that post-treatment EEG abnormalities, including BASED score and residual epileptiform burden, may be associated with relapse after initial response (13, 15). In this cohort, children with sustained 6-month spasm freedom showed robust post-treatment suppression of ripple propagation networks, whereas children who later relapsed showed incomplete suppression. This finding raises the possibility that residual ripple propagation after apparent clinical response reflects incomplete control of pathological epileptic network activity. However, the relapse analysis was limited by only seven relapse events, lack of external validation, and a cutoff derived from the same cohort. Therefore, residual ripple propagation should not be interpreted as a clinically actionable relapse predictor at this stage, but rather as a candidate marker for prospective testing.

### 4.5 Implications for neuroimmune treatment monitoring in IESS

The present study provides a potential bridge between ACTH-related neuroendocrine–immunomodulatory therapy and objective electrophysiological monitoring. Direct immune biomarkers may reveal upstream biological responses, whereas scalp HFO propagation metrics may capture downstream network-level consequences of treatment (2, 40–42). This distinction is important because successful immunomodulatory or neuroendocrine therapy ultimately needs to suppress the pathological neuronal network responsible for spasms. Scalp ripple propagation network analysis may therefore complement immune, endocrine, and conventional EEG biomarkers in future multimodal models of treatment response.

### 4.6 Methodological considerations

Scalp HFO analysis has important limitations, including vulnerability to muscle artifacts, filtering artifacts, sharp transients, and volume conduction (44–46). The present study attempted to reduce these confounds by selecting artifact-free slow-wave sleep segments, using a standardized preprocessing pipeline, applying bipolar montage, and focusing on non-zero-lag time-delay propagation. Nevertheless, the specificity of automatic ripple detection remains imperfect, and the 250-ms propagation window may capture some chance co-occurrences. Future studies should incorporate manual validation of detected ripples, time-shuffled surrogate analyses, sensitivity analyses using alternative propagation windows, and high-density EEG or source-localized recordings. These methodological safeguards are particularly important if scalp HFO propagation metrics are to be used as response-verification biomarkers rather than descriptive research measures.

### 4.7 Limitations

Several limitations should be acknowledged. First, the retrospective design may introduce selection bias and requires prospective validation. Second, the sample size was modest, particularly for relapse analysis. Third, inflammatory cytokines, peripheral immune-cell profiles, blood–brain barrier or neurovascular-unit markers, and endocrine biomarkers were not systematically available; therefore, this study cannot establish a direct immunological mechanism. Fourth, standardized BASED scoring and systematic hypsarrhythmia classification were not available for all participants, limiting direct comparison with conventional EEG severity markers (13, 15). Fifth, center effects, including EEG systems, acquisition conditions, ACTH dosing, and clinical practice, were not fully adjusted. Sixth, standard 10–20 scalp EEG provides limited spatial resolution, and volume conduction remains a concern (45, 46). Seventh, only 5-minute slow-wave sleep segments were analyzed, which may not capture the full temporal variability of HFO burden or propagation. Eighth, the prespecified 250-ms window may include chance co-occurrences or associations produced by common network input. Therefore, the resulting directed edges represent candidate temporal relationships rather than confirmed physiological propagation. Finally, developmental outcomes were not included. Future prospective studies should integrate HFO propagation metrics with standardized EEG background assessment, immune and endocrine biomarkers, neuroimaging, and long-term neurodevelopmental follow-up.

## 5 Conclusions

Effective ACTH-related neuroendocrine–immunomodulatory therapy in infantile epileptic spasms syndrome was associated with suppression of scalp ripple propagation network strength, suggesting that directed high-frequency network activity may serve as a downstream electrophysiological readout of treatment response. The dissociation between reduced ripple counts and persistent network connectivity in nonresponders indicates that ripple burden and putative ripple propagation network organization capture related but non-identical aspects of pathological high-frequency activity. Exploratory relapse analyses further suggest that residual ripple propagation after apparent acute response may be relevant to early recurrence, although this finding requires validation in larger prospective cohorts. These results support scalp ripple propagation analysis as a complementary marker of adrenocorticotropic hormone-related network suppression and provide a rationale for future studies combining electrophysiological network measures with standardized EEG background assessment, neuroendocrine and inflammatory biomarkers, and long-term developmental follow-up.

## Data Availability

Deidentified data supporting the findings of this study may be made available from the corresponding authors upon reasonable request and subject to institutional ethics approval and data-sharing regulations. Requests to access these datasets should be directed to Xiaofeng Yang, xiaofengyang@yahoo.com.
