# Supplementary material for: Electrophysiological readout of ACTH-related neuroendocrine–immunomodulatory treatment response in infantile epileptic spasms syndrome: suppression of scalp ripple propagation networks
Source: Front Immunol. 2026 Aug 10;17:1923197. doi: 10.3389/fimmu.2026.1923197 (PMC13500334; doi:10.3389/fimmu.2026.1923197)
Supplement: Supplementary file 1 [file DataSheet1.docx]

**Supplementary Material:**

**Figure S1.** **Flowchart of patient selection criteria.**

**Table S1. Diagnostic performance of ripple count and the primary network metrics at optimal cut-off points for evaluating therapy response post-ACTH therapy.**

| Index | total NCS | RR of total NCS | ripple count | RR of ripple count |
| --- | --- | --- | --- | --- |
| AUC | 0.902  (0.799-0.963) | 0.899  (0.794-0.961) | 0.821  (0.702-0.907) | 0.769  (0.643-0.867) |
| Cut-off value | 13.4 | 0.642 | 170 | 0.831 |
| Sensitivity | 0.941  (0.809,0.984) | 0.941  (0.809,0.984) | 0.794  (0.632,0.897) | 0.941  (0.809,0.984) |
| Specificity | 0.852  (0.675,0.941) | 0.815  (0.633,0.918) | 0.889  (0.719,0.961) | 0.704  (0.515,0.841) |
| Accuracy | 0.902  (0.802,0.954) | 0.885  (0.782,0.943) | 0.836  (0.724,0.908) | 0.836  (0.724,0.908) |
| YI | 0.793 | 0.756 | 0.683 | 0.645 |
| PPV | 0.889  (0.747,0.956) | 0.865  (0.720,0.941) | 0.900  (0.744,0.965) | 0.800  (0.652,0.895) |
| NPV | 0.920  (0.750,0.978) | 0.917  (0.742,0.977) | 0.774  (0.602,0.886) | 0.905  (0.711,0.973) |
| PLR | 5.78  (2.47,13.5) | 4.73  (2.22,10.1) | 6.29  (2.32,17.0) | 3.06  (1.73,5.40) |
| NLR | 0.085  (0.026,0.284) | 0.089  (0.027,0.298) | 0.245  (0.128,0.469) | 0.103  (0.030,0.347) |

Note: 95% confidence intervals are reported in parentheses. YI, Youden's Index; PPV, Positive Predictive Value; NPV, Negative Predictive Value; PLR, Positive Likelihood Ratio; NLR, Negative Likelihood Ratio; AUC, area under the curve; NCS, network connection strength; RR, reduction ratio.

**Table S2. Diagnostic performance of the secondary network metrics at optimal cut-off points for evaluating therapy response post-ACTH therapy.**

| Index | mean NCS | RR of mean NCS | number of network nodes | RR of number of network nodes |
| --- | --- | --- | --- | --- |
| AUC | 0.872  (0.762-0.944) | 0.829  (0.711-0.913) | 0.861  (0.749-0.936) | 0.799  (0.677-0.891) |
| Cut-off value | 0.926 | 0.553 | 11.5 | 0.252 |
| Sensitivity | 1.00  (0.898,1.00) | 0.941  (0.809,0.984) | 0.941  (0.809,0.984) | 1.00  (0.898,1.00) |
| Specificity | 0.667  (0.478,0.814) | 0.667  (0.478,0.814) | 0.741  (0.553,0.868) | 0.667  (0.478,0.814) |
| Accuracy | 0.852  (0.743,0.920) | 0.820  (0.705,0.896) | 0.852  (0.743,0.920) | 0.852  (0.743,0.920) |
| YI | 0.667 | 0.608 | 0.682 | 0.667 |
| PPV | 0.791  (0.648,0.886) | 0.780  (0.633,0.880) | 0.821  (0.673,0.910) | 0.791  (0.648,0.886) |
| NPV | 1.00  (0.824,1.00) | 0.900  (0.699,0.972) | 0.909  (0.722,0.975) | 1.00  (0.824,1.00) |
| PLR | 2.91  (1.73,4.88) | 2.74  (1.62,4.63) | 3.47  (1.87,6.44) | 2.91  (1.73,4.88) |
| NLR | NA | 0.108  (0.032,0.368) | 0.098  (0.029,0.329) | NA |

Note: 95% confidence intervals are reported in parentheses. YI, Youden's Index; PPV, Positive Predictive Value; NPV, Negative Predictive Value; PLR, Positive Likelihood Ratio; NLR, Negative Likelihood Ratio; AUC, area under the curve; NCS, network connection strength; RR, reduction ratio.

**Table S3. Pairwise Comparisons of ROC Curves for Ripple Count Metrics and Ripple Network Metrics Using the DeLong Test (evaluating therapy response).**

| Index 1 | Index 2 | △AUC | 95% CI | Z value | p | Adjusted P |
| --- | --- | --- | --- | --- | --- | --- |
| ripple count | total NCS | 0.081 | -0.004 - 0.166 | 1.876 | 0.061 | 0.121 |
|  | RR of total NCS | 0.078 | -0.023 - 0.178 | 1.518 | 0.129 | 0.129 |
|  | number of network nodes | 0.040 | -0.045 - 0.126 | 0.924 | 0.356 | 0.712 |
|  | RR of number of network nodes | 0.022 | -0.119 - 0.163 | 0.303 | 0.762 | 0.902 |
|  | mean NCS | 0.051 | -0.050 - 0.152 | 0.993 | 0.321 | 0.712 |
|  | RR of mean NCS | 0.008 | -0.122 - 0.138 | 0.123 | 0.902 | 0.902 |
| RR of ripple count | total NCS | 0.133 | 0.010 - 0.257 | 2.111 | **0.035** | **0.035** |
|  | RR of total NCS | 0.130 | 0.027 - 0.233 | 2.472 | **0.014** | **0.027** |
|  | number of network nodes | 0.093 | -0.026 - 0.212 | 1.526 | 0.127 | 0.280 |
|  | RR of number of network nodes | 0.031 | -0.084 - 0.145 | 0.523 | 0.601 | 0.601 |
|  | mean NCS | 0.103 | -0.034 - 0.241 | 1.474 | 0.140 | 0.280 |
|  | RR of mean NCS | 0.061 | -0.074 - 0.194 | 0.884 | 0.377 | 0.503 |

Note: Raw P values and adjusted P values are presented. Multiple comparisons were corrected using the Holm procedure for primary confirmatory comparisons and the Benjamini–Hochberg procedure for secondary exploratory comparisons. Bolded numbers indicate p-values < 0.05.

AUC, area under the curve; NCS, network connection strength; RR, reduction ratio.

**Table S4. Diagnostic performance of ripple count and network metrics at optimal cut-off points for predicting six-month outcome post-ACTH therapy.**

| Index | total NCS | mean NCS | RR of total NCS | RR of mean NCS | RR of ripple count |
| --- | --- | --- | --- | --- | --- |
| AUC | 0.807  (0.610-0.932) | 0.821  (0.627-0.941) | 0.943  (0.781-0.996) | 0.864  (0.678-0.965) | 0.829  (0.635-0.945) |
| Cut-off value | 16.7 | 1.59 | 0.661 | 0.509 | 0.673 |
| Sensitivity | 0.571  (0.250,0.842) | 0.571  (0.250,0.842) | 0.857  (0.487,0.974) | 0.857  (0.487,0.974) | 0.714  (0.359,0.918) |
| Specificity | 1.00  (0.839,1.00) | 0.950  (0.764,0.991) | 1.00  (0.839,1.00) | 0.850  (0.640,0.948) | 0.850  (0.640,0.948) |
| Accuracy | 0.889  (0.719,0.961) | 0.852  (0.675,0.941) | 0.963  (0.817,0.993) | 0.852  (0.675,0.941) | 0.815  (0.633,0.918) |
| YI | 0.571 | 0.521 | 0.857 | 0.707 | 0.564 |
| PPV | 1.00  (0.510,1.00) | 0.800  (0.376,0.964) | 1.00  (0.610,1.00) | 0.667  (0.354,0.879) | 0.625  (0.306,0.863) |
| NPV | 0.870  (0.679,0.955) | 0.864  (0.667,0.953) | 0.952  (0.773,0.992) | 0.944  (0.742,0.990) | 0.895  (0.686,0.971) |
| PLR | NA | 7.88  (1.50,41.4) | NA | 4.88  (1.77,13.4) | 4.13  (1.42,12.0) |
| NLR | 0.448  (0.204,0.986) | 0.471  (0.213,1.04) | 0.192  (0.045,0.814) | 0.225  (0.053,0.964) | 0.375  (0.132,1.07) |

Note: 95% confidence intervals are reported in parentheses. When the denominator is 0 in the calculation of the PLR, it is usually because the specificity is 1 (there is no false positive), and the valid value cannot be obtained mathematically.

YI, Youden's Index; PPV, Positive Predictive Value; NPV, Negative Predictive Value; PLR, Positive Likelihood Ratio; NLR, Negative Likelihood Ratio; AUC, area under the curve; NCS, network connection strength; RR, reduction ratio.

**Table S5. Results of pairwise comparisons of ROC curves for ripple count metrics and ripple network metrics using the DeLong test (predicting six-month outcome).**

| Index 1 | Index 2 | △AUC | 95% CI | Z value | p | Adjusted P |
| --- | --- | --- | --- | --- | --- | --- |
| RR of ripple count | total NCS | 0.022 | -0.190-0.233 | 0.199 | 0.842 | 0.842 |
|  | RR of total NCS | 0.114 | -0.042-0.271 | 1.429 | 0.153 | 0.306 |
|  | mean NCS | 0.007 | -0.220-0.235 | 0.062 | 0.951 | 0.951 |
|  | RR of mean NCS | 0.036 | -0.176-0.248 | 0.330 | 0.742 | 0.951 |

Note :Raw P values and adjusted P values are presented. Multiple comparisons were corrected using the Holm procedure for primary confirmatory comparisons and the Benjamini–Hochberg procedure for secondary exploratory comparisons. Adjusted P values <0.05 were considered statistically significant. AUC, area under the curve; NCS, network connection strength; RR, reduction ratio.
